# Detection and Quantification of Milk Ingredients as Hidden Allergens in Meat Products by a Novel Specific Real-Time PCR Method

**DOI:** 10.3390/biom9120804

**Published:** 2019-11-29

**Authors:** Caterina Villa, Joana Costa, Isabel Mafra

**Affiliations:** REQUIMTE-LAQV, Faculdade de Farmácia, Universidade do Porto, Rua de Jorge Viterbo Ferreira, 228, 4050-313 Porto, Portugal; caterinavilla@hotmail.com

**Keywords:** food allergen, real-time PCR, 12S rRNA gene, quantification, meat products, milk

## Abstract

Milk ingredients are often included in a wide range of meat products, such as cooked hams and sausages, to improve technological characteristics. However, milk proteins are also important food allergens. The aim of this study was the development of a highly sensitive and specific real-time PCR system targeting the 12S rRNA gene of *Bos domesticus* for the detection and quantification of milk as an allergenic ingredient in processed meat products. The method was able to achieve an absolute limit of detection (LOD) of 6 fg of milk DNA. Using a normalized approach (∆Ct method) for the detection of milk protein concentrate (MPC), it was possible to obtain sensitivities down to 0.01% (w/w) of MPC in model hams (raw and cooked) and autoclaved sausages, and 0.005% in raw sausage mixtures. The developed systems generally presented acceptable PCR performance parameters, being successfully validated with blind samples, applied to commercial samples, and further compared with an immunochemical assay. Trace amounts of milk material were quantified in two out of 13 samples, but the results mostly infer the excessive practice of the precautionary labeling.

## 1. Introduction

The meat industry uses a wide range of ingredients with specific technological properties, to improve the appearance, taste and texture of products, as well as their nutritional value. In some cases, the introduction of these ingredients is intended to decrease the production costs or is simply unintentional introduced due to bad manufacturing practices. However, some of these ingredients are a source of major food allergens, such as milk, legumes, egg, celery or cereal gluten proteins, causing a high health risk to consumers suffering from food allergies [1]. Milk proteins are often added to meat products in order to improve their juiciness, texture, and flavor. These proteins can be caseins or whey proteins, such as ß-lactoglobulin and α-lactalbumin, which are considered major food allergens. Milk allergy is one of the most common food allergies in early childhood that often tends to resolve with age, although it can also persist through adulthood [2]. People with allergies are forced to implement a complete milk elimination diet in order to avoid adverse reactions, which can vary from mild symptoms, affecting the respiratory or the gastrointestinal tract, to severe symptoms leading to anaphylaxis [3]. To guarantee the safety of 95% of milk-allergic patients, a threshold of clinical reactivity to milk of 30 mg/kg was established by Morisset et al. [4], based on the consumption of 100 g of product. Considering the eliciting dose that protects 99% of the milk-allergic population (ED01), a new reference dose was recently defined as 0.1 mg of milk proteins using proper statistical dose-distribution models. This value represents 3.03 mg of liquid milk per kg of food and 0.28 mg of non-dry fat milk per kg of food, according to the conversion factors available from the U.S. Department of Agriculture [5]. Therefore, analytical methods able to detect and quantify trace amounts of milk allergens in processed foodstuffs are needed to identify their presence as ingredients or as cross-contaminants and verify labeling compliance.

Recently, DNA-based methods have been revealed as promising techniques in the field of food allergen detection, due to the great thermal stability of DNA molecules as compared with proteins, especially upon severe food processing conditions. When analyzing processed foods, such as meat products, polymerase chain reaction (PCR) techniques can be very useful alternatives to immunochemical methods [6]. Until now, the detection of milk allergens in foodstuffs has been mostly performed by protein analysis, relying on mass spectrometry [7,8,9,10,11] and ELISA [12,13,14,15]. However, the application of PCR-based methods to detect and quantify milk as an allergenic food is still limited. Xiao et al. [16] performed a real-time PCR assay using a TaqMan minor groove binder probe to recognize the α-lactalbumin gene from the cow. However, the quantitative aptitude of the method was limited since they only quantified the cow’s milk DNA, with a rough estimation of milk content as an ingredient and without showing any calibration curve. In such cases, the use of reference materials or model mixtures is crucial to develop quantitative calibration models for allergen analysis [17]. Moreover, the use of a normalized calibration curve is highly recommended to account for possible amplification differences due to inconsistent DNA recovery and quality and degradation among extracts as a result of food processing [6,18].

Previous to this work, Villa et al. [19] performed an extensive evaluation of molecular markers to assess their suitability for the development of a specific real-time PCR method to detect and quantify milk ingredients in cooked hams. The results highlighted the use of a fragment targeting the 12S rRNA gene of *Bos domesticus* as the most specific and sensitive marker. On the basis of such preliminary findings, this study intends to develop a specific and sensitive normalized real-time PCR assay targeting the 12S rRNA gene to detect and quantify milk protein concentrates in meat products. Additionally, it is intended to evaluate the effects of food matrix and processing using model mixtures, simulating the preparation of cooked hams and sausages (oven cooking for hams and autoclaving for sausages). Finally, the validation of the method with blind mixtures and its further application to analyze commercial meat products was performed to verify their labeling compliance, with the results being further compared with ELISA.

## 2. Materials and Methods

### 2.1. Reference Model Mixtures

In the absence of certified reference or testing materials for the specific detection of cow’s milk powders in meat products, model mixtures of turkey meat spiked with known amounts of cow’s milk protein concentrate (MPC) were prepared. For this study, two independent sets of model mixtures containing 10.0%, 5.0%, 1.0%, 0.5%, 0.1%, 0.05%, 0.01%, 0.005%, 0.001%, 0.0005%, and 0.0001% (w/w) of MPC in minced turkey meat were prepared simulating cooked hams and sausages. Turkey meat was previously minced using a laboratory knife mill (Grindomix GM200, Retsch, Haan, Germany). To simulate ham preparation, 8 g of salt and 4 g of powder sugar were added to 1 kg of meat, while the sausages were prepared, adding 250 g of ice, 20 g of salt, and 375 g of lard to 500 g of turkey meat. To facilitate homogenization, 10 mL of a sterile phosphate-buffered saline solution (0.2 M) was added to both mixtures. The turkey meat (muscle) was acquired at a local retail market (Porto, Portugal) and the MPC was provided by a food additive company (Formulab, Maia, Portugal). The exact protein content of MPC was determined by the Kjeldahl method, corresponding to 83.4% of total milk protein. Accordingly, taking into consideration this value, first mixtures containing 10% of MPC were prepared by adding the required amount of MPC to the ham or sausage raw mixtures (in a total of 200 g).

Each of the two sets of mixtures containing MPC, prepared according to ham and sausage recipes, was divided into two subsets, from which one was immediately stored at −20 °C, while the other was subjected to the respective thermal processing (described in Section 2.3).

To avoid contaminations, all mixtures and meat samples were homogenized separately, and all materials and different containers were previously treated with DNA decontamination solution.

### 2.2. Validation Mixtures and Commercial Samples

For method validation, two sets of blind mixtures, for hams and sausages, were prepared similarly to the reference mixtures, in order to contain 4.0%, 0.8%, 0.4%, 0.2%, and 0.002% (w/w) of MPC. Similar to the reference mixtures, both sets of blind mixtures were also divided into two subsets and submitted to the respective thermal treatment. Additionally, 13 commercial food samples of cooked hams (n = 6) and sausages (n = 7), acquired at local markets, were used to assess the applicability of the method. Validation mixtures and commercial samples were homogenized separately in a laboratory knife mill Grindomix GM200 (Retsch, Haan, Germany), using different containers and knives previously treated with DNA decontamination solution and immediately stored at −20 °C after preparation until DNA extraction.

### 2.3. Thermal Treatments

Two distinct thermal treatments were applied to the subsets of reference and validation mixtures to evaluate the effect of thermal processing. For cooked ham simulation, the mixtures were oven cooked at 67 °C for 5 h, whereas for sausage simulation, the mixtures were autoclaved (121 °C, 1 bar) for 15 min. The thermally treated mixtures were immediately stored at −20 °C until DNA extraction.

### 2.4. DNA Extraction

The NucleoSpin food kit (Macherey-Nagel, Düren, Germany), with some minor modifications according to the manufacturer’s instructions, was used to extract the DNA from the reference and validation model mixtures, as well as from commercial samples, with the addition of 2 μL of RNase (2 mg/mL) after the cell lysis step. The extractions were performed at least in duplicate assays using 200 mg of each sample. The extracts were kept at −20 °C until further analysis.

In order to evaluate the yield and purity of DNA extracts, UV spectrophotometric DNA quantification was performed on a SynergyHT multimode microplate reader (BioTek Instruments, Inc., Winooski, VT, USA), using a Take 3 micro-volume plate accessory. The DNA content was assessed by the nucleic acid quantification protocol with sample type defined for double-strand DNA in the Gen5 data analysis software version 2.01 (BioTek Instruments, Inc., Winooski, VT, USA).

### 2.5. Oligonucleotide Primers and Probes

The oligonucleotide primers and probes used in this work were synthesized by Eurofins MWG Operon (Ebersberg, Germany) and they are presented in Table 1. For the specific detection of bovine milk, primers (916/916-R) and hydrolysis probe (916-P) targeting the bovine region of 12S rRNA gene with NCBI accession no. AY526085.1 were retrieved from [19]. Primers (EUK-F/EUK-R) and hydrolysis probe (S5), designed in the conserved region of the 18S rRNA gene, were used to target a universal eukaryotic sequence of 120 pb as an endogenous control [20]. To evaluate the amplification capacity of extracts, all the samples were amplified by qualitative PCR using 18SRG-F/18SRG-R primers retrieved from Costa et al. [21] targeting a sequence of 113 bp in the same conserved eukaryotic gene (NCBI accession no. HQ873432.1).

### 2.6. Qualitative PCR

The PCR amplifications were performed in a MJ Mini^TM^ Gradient Thermal Cycler (Bio-Rad Laboratories, Hercules, CA, USA) in a total reaction volume of 25 µL containing 2 µL of template DNA (40 ng), 1× buffer (67 mM of Tris-HCl (pH 8.8), 16 mM of (NH_4_)_2_SO_4_, 0.1% of Tween 20), 200 μM of each dNTP (Grisp, Porto, Portugal), 1.0 U of SuperHot Taq DNA Polymerase (Genaxxon Bioscience, Ulm, Germany), 3.0 mM or 1.5 mM of MgCl_2_ for 916/916-R and 18SRG-F/18SRG-R primers, respectively, and 200 nM or 240 nM of each primer, 916/916-R and 18SRG-F/18SRG-R, respectively (Table 1). The amplification programs were defined as follows: initial denaturation at 95 °C for 5 min; 40 cycles (for primers 916/916-R) or 33 (18SRG-F/18SRG-R) at 95 °C for 30 s, 56 °C (916/916-R) or 65 °C (18SRG-F/18SRG-R) for 30 s and 72 °C for 30 s; and a final extension at 72 °C for 5 min.

PCR products were verified by electrophoresis in a 1.5% agarose gel stained with GelRed 1× (Biotium, Inc., Hayward, CA, USA) and carried out in 1× SGTB (Grisp, Porto, Portugal) for 25 to 30 min at 200 V. Agarose gel visualization was performed in a UV light tray Gel Doc™ EZ System (Bio-Rad Laboratories, Hercules, CA, USA), recording a digital image with Image Lab software version 5.2.1 (Bio-Rad Laboratories, Hercules, CA, USA). Each extract was amplified at least in two independent runs.

### 2.7. Real-Time PCR

For the real-time PCR amplifications, the reaction mixture of 20 µL included 1× SsoFast Probes Supermix (Bio-Rad Laboratories, Hercules, CA, USA), 240 nM of each primer (916/916-R or EUK-F/EUK-R), 160 nM of each probe (916-P or S5) (Table 1) and 2 µL of DNA extract (40 ng). Each target sequence (eukaryotic and 12S rRNA genes) was amplified in parallel reactions and run simultaneously in a fluorometric thermal cycler CFX96 real-time PCR detection system (Bio-Rad Laboratories, Hercules, CA, USA) with the following conditions: 95 °C for 5 min, 50 cycles at 95 °C for 10 s, 56 °C for 10 s, and 72 °C during 30 s, with collection of fluorescence signal at the end of each cycle. The software Bio-Rad CFX Manager 3.1 (Bio-Rad Laboratories, Hercules, CA, USA) was used to evaluate the data from each real-time PCR run. Cycle of quantification (Cq), also known as cycle threshold (Ct), values were calculated using the software at automatic threshold settings. Real-time PCR trials were repeated in two or three independent runs using n = 4 replicates in each one.

### 2.8. ELISA

To validate the quantitative results of analyzed commercial samples by real-time PCR, the RIDASCREEN^®^ FAST Milk ELISA kit was used (R-Biopharm AG, Darmstadt, Germany). This is a sandwich enzyme immunoassay to quantify milk proteins in food using antibodies to specifically detect caseins and β-lactoglobulin of cow’s milk in the range of 0 to 67.5 mg/kg. The assay was carried out according to the manufacturer’s instructions. Based on the quantitative real-time PCR results, samples #6 and #12 were appropriately diluted (1000-fold and 5-fold, respectively) to ensure their analysis within the range of the calibration curve of the kit. All the remaining samples were not diluted. The absorbency values were plotted against the concentration of standard solutions containing milk proteins. A nonlinear regression function was carried out using a sigmoid four parametric logistic function based on the following expression:

$$Y=\frac{A-D}{1+{\left(\frac{X}{C}\right)}^{b}}+D$$

where Y is the optical density (absorbance), A the maximum absorbance, b the slope of the calibration curve in linear range, C the 50% inhibitory concentration (IC50) (μg/L), D the minimum absorbance, and X the analyte concentration (μg/L). Each sample was analyzed in triplicate.

### 2.9. Statistical Analysis

The statistical analysis was performed using the software IBM SPSS Statistics (23.0 package, IBM Corporation, New York, NY, USA). The significance of differences between the ΔCt values of reference mixtures at the same spiking level was performed using independent samples t-test. Significant differences were considered when *p* < 0.05.

## 3. Results and Discussion

### 3.1. Development of the Analytical Method

Meat products usually have a very complex composition, containing not only meat and fat, but also several other ingredients that improve their technological properties, nutritional value or flavor. MPC is one of the ingredients often added to meat products [1], but since it is also an allergenic food source, its addition poses a concrete health risk for the milk-allergic individuals. The development of specific and sensitive methods that are able to detect and quantify this type of additive is of general concern, aiming at fulfilling the growing expectations of a global market in providing safe food to consumers. In the specific case of meat products that undergo different processing treatments (thermal, radiation, high pressure or fermentation) during their production, allergenic proteins can be exposed to structural and physical and chemical modifications affecting their detection by immunochemical methods [6,18]. Therefore, DNA-based methods have been applied as reliable alternatives to immunochemical assays for allergen detection. Despite being considered as indirect approaches for allergen analysis, PCR-based methods can enable the detection of specific DNA sequences as unequivocal taxon marker molecules, generally down to the species level. In addition, with the use of appropriate reference materials as calibrants and real-time PCR, the presence of an allergenic species can be estimated. Although real-time PCR does not allow the identification of any specific protein (or allergen), it enables quantifying the amount of an allergenic food within a complex matrix, which is crucial information to verify labeling compliance and the safety of foods [6,18,20,21]. Therefore, a positive DNA result can be directly correlated with the presence of proteins from the allergenic food source. In certain cases where the amount of amplifiable DNA is limited, the application of PCR-based methods might not be possible. Nevertheless, this could be overcome with the use of appropriate DNA extraction methods, taking advantage of the high sensitivity in detecting trace amounts of degraded DNA. This has been demonstrated in our previous work in which DNA was extracted from milk products [19]. In this case, the choice of the DNA extraction method was crucial to obtain successful amplification results. Accordingly, the NucleoSpin food kit (Macherey-Nagel, Düren, Germany), with the addition of RNase after the cell lysis step, was chosen from our previous results from milk DNA extraction, which provided the best DNA yields, purities, and amplification results [19].

**Figure 1:**
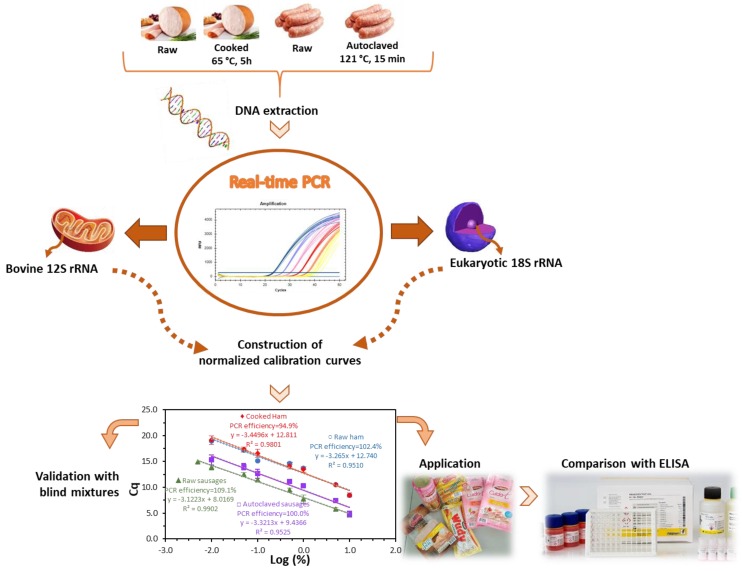
Schematic diagram representing the developed work.

Following the successful DNA extraction, an extensive screening study on mitochondrial and nuclear bovine genes was performed to identify the best candidate markers for MPC detection in processed meat products [19]. The study revealed that it is a challenging task to obtain a sensitive and specific DNA method to detect milk proteins at trace levels. On the one hand, the high similarity among mitochondrial sequences of mammal and avian species interferes when high sensitivity levels are implemented for allergen analysis. On the other hand, the nuclear allergen-encoding genes provide low sensitivity assays, not suitable for allergen analysis. The sequence of the 12S rRNA gene provided the best compromise of sensitivity and specificity, being the selected marker to develop a quantitative method for MPC detection in meat products. In this work, the specific primers and the hydrolysis TaqMan probe previously designed were used to develop a normalized real-time PCR system with raw and heat-treated model mixtures of turkey meat spiked with known amounts of MPC. To evaluate the effect of food matrix and processing, two sets of mixtures were prepared, one simulating cooked ham and the other autoclaved sausage production. At a first stage, a calibration curve for absolute DNA detection of MPC was obtained, followed by the development of four normalized calibration curves for the relative quantification based on the parallel amplification of two sequences, i.e., the bovine target 12S rRNA gene and a universal eukaryotic gene as endogenous control. The method validation was performed using blind mixtures and according to the general guidelines adopted for this type of assay [22,23], since no official requirements are yet defined for allergen testing based on DNA analysis. The validated method was applied to commercial samples and data were further compared with the results of the application of an immunochemical assay. A schematic diagram representing the workflow of this study is presented in Figure 1.

#### 3.1.1. Absolute and Relative Sensitivity

**Figure 2:**
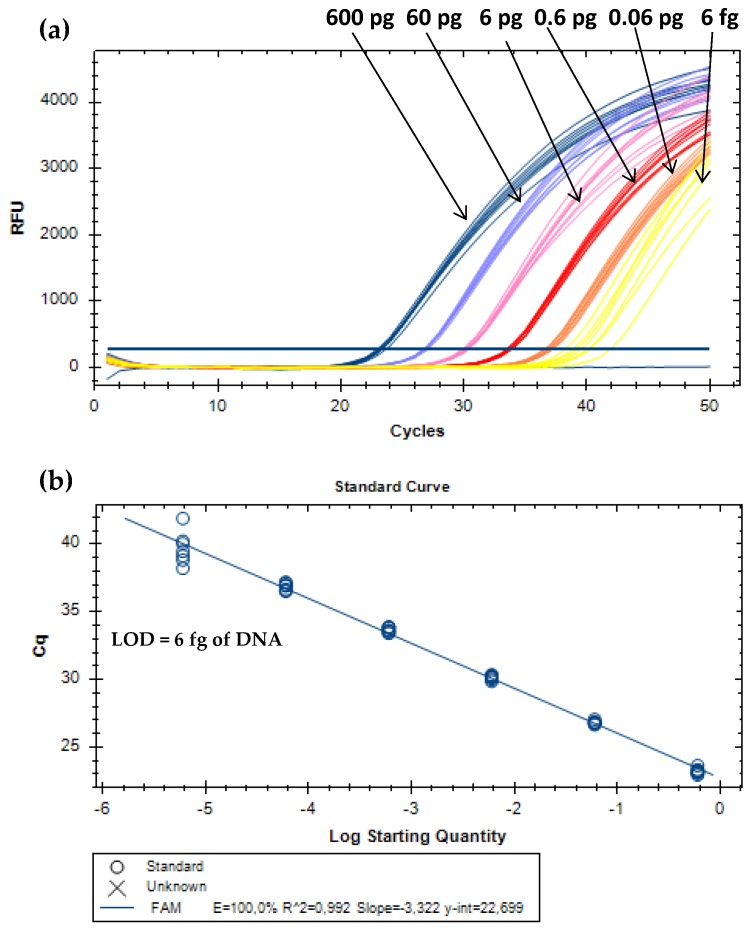
Amplification curves (**a**) and respective calibration curve (**b**) obtained by real-time PCR with TaqMan probe targeting the 12S rRNA gene using serially diluted (1/10) DNA extracts of MPC from 0.6 ng to 6 fg (*n* = 8 replicates).

**Figure 3:**
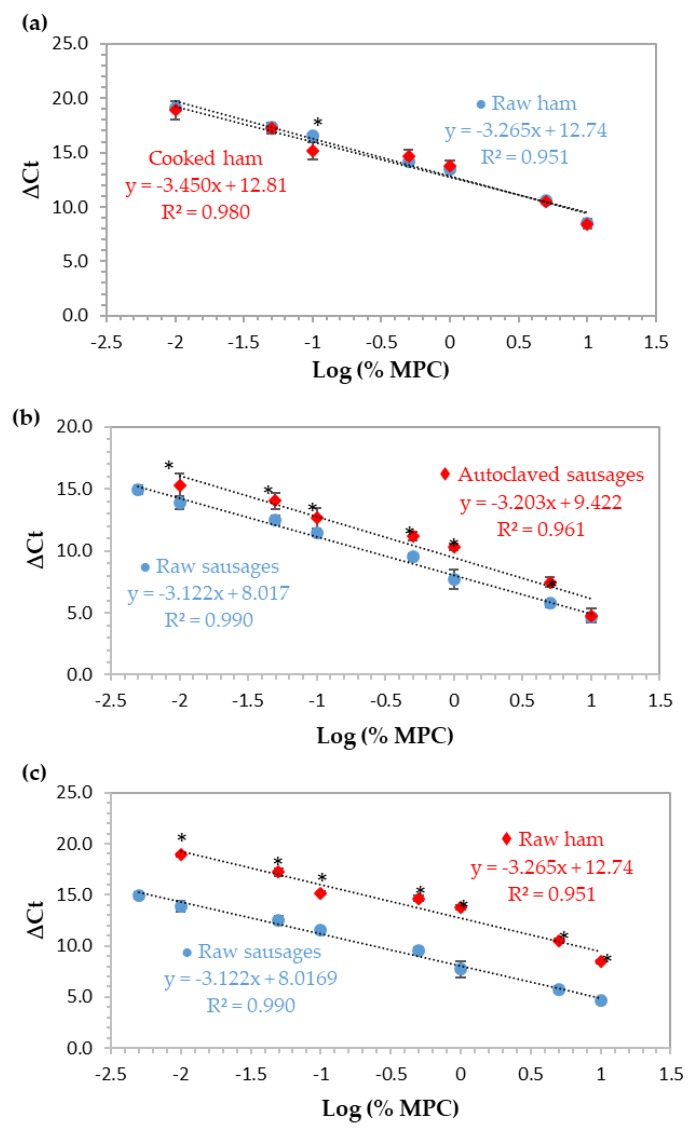
Normalized calibration curves obtained by real-time PCR with TaqMan probe targeting the 12S rRNA gene using ham and sausage model mixtures with 10%, 5%, 1%, 0.5%, 0.1%, 0.05%, 0.01%, and 0.005% (w/w) of MPC (*n* = 8 replicates) with and without thermal treatment. (**a**) Raw and cooked ham reference mixtures, (**b**) raw and autoclaved sausage mixtures, and (**c**) raw mixtures of hams and sausages. * Significant differences (*p* < 0.05) between the ΔCt values at the same spiking level (independent samples t-test).

For absolute detection, DNA extracts of MPC were 10-fold serially diluted to cover six orders of magnitude of the target analyte (from 0.6 ng to 0.6 fg of bovine DNA). The proposed real-time PCR assay enabled the amplification of all replicates (eight/eight) until the level of 6 fg of milk (Figure 2). Therefore, this value was considered the absolute limit of detection (LOD), which is defined as the lowest concentration level of the analyte with positive amplification at least 95% of the times, according to the parameters required for method development and validation [22,23]. Other performance parameters have to comply with the acceptance criteria established for real-time PCR assays. The PCR efficiency should be between 90% and 110%, the correlation coefficient (R^2^) equal or above 0.98, and the slope within −3.6 and −3.1 [22,23]. The calibration curve obtained for absolute quantification (Figure 2) is in agreement with these criteria, with a PCR efficiency of 100.0%, a slope of –3.322, and a R^2^ of 0.99. For the relative detection, each subset of model mixtures containing known amounts of MPC (10.0%, 5.0%, 1.0%, 0.5%, 0.1%, 0.05%, 0.01%, 0.005%, 0.001%, 0.0005%, and 0.0001%, w/w) in turkey meat, simulating cooked ham and sausages, before and after thermal treatment, were used. All the real-time PCR systems enabled dynamic ranges of four orders of magnitude, with amplifications down to 0.01% (w/w) (100 mg/kg) of MPC in both subsets of hams (raw and cooked) and autoclaved sausages, whereas in raw sausage mixtures it was down to 0.005% (w/w) (50 mg/kg) of MPC. Therefore, both values were considered as relative LOD for the respective systems since all the replicates were amplified (Table 2, Figure 3).

Most studies using DNA-based methods targeting mitochondrial multi-copy genes focus on the authentication of milk and milk products, with sensitivities above 0.1% of cow DNA, not enough in the field of allergen detection [24]. The real-time PCR method developed by Xiao et al. [16] showed an absolute sensitivity of 0.05 ng of bovine DNA, being applied to 42 commercial food samples. However, the authors did not use reference mixtures as calibrants to develop an effective quantitative approach. 

Köppel et al. [25,26] reached an absolute sensitivity down to 0.64 μg/mL of bovine DNA, targeting the mitochondrial bovine tRNA-Lys gene, for the detection of cow’s milk in processed foods as an allergen, but without its quantification. To the best of our knowledge, the proposed real-time PCR assay enables the highest sensitivity, both absolute and relative, for the detection of cow’s milk ingredients in complex food products, enabling its correct estimation by the use of reference mixtures.

#### 3.1.2. Construction and Validation of the Normalized Quantitative Model

For the relative quantification of MPC in cooked ham and sausages, a normalized curve based on the ∆Ct method was constructed for each type and set of model mixtures (ham/sausage and raw/processed). This approach uses the cycle threshold (Ct) values obtained from the parallel amplification of the target sequence (12S rRNA gene of cow) and a universal eukaryotic region (18S rRNA gene as endogenous control) with approximately the same amplification efficiencies, in order to assure the accuracy of results [22]. In this study, ∆Ct values were obtained by the difference between the Ct values of cow and eukaryotic amplifications and plotted against the logarithm of MPC percentage of eight concentration levels (10%, 5%, 1%, 0.5%, 0.1%, 0.05%, 0.01%, and 0.005%, w/w). The obtained calibration curves, each one constructed with the mean values of two independent real-time PCR runs (n = 8), are presented in Figure 3 and the respective PCR performance parameters are summarized in Table 2. All the results are in agreement with the accepted criteria defined for this type of assay [22,23], except for the correlation coefficient of the curves of raw ham and autoclaved sausages (0.951 and 0.961, respectively). During the preparation of ham mixtures, retention of a small quantity of water when they were raw was noticed, which was eliminated after thermal treatment and could affect DNA extraction and PCR amplifications, thus, contributing to a low R^2^ value. In the case of autoclaved sausages, the severe thermal treatment might be the main reason to affect this parameter negatively.

In our previous work describing a preliminary real-time PCR trial using known amounts of MPC in raw turkey meat and cooked hams as model mixtures, but without normalization, the Ct values above the threshold of cross-species amplification (38 cycles) were considered negative, setting a relative LOD of 0.05% (500 mg/kg, w/w) of MPC in raw and cooked ham [19]. Comparing the present results with the previous findings, the relative sensitivity increased to 0.01% (100 mg/kg, w/w) in both ham model mixtures, even considering the 38-cycle limit above which the amplification is unreliable due to cross-reactivity with other species, namely chicken and goat [19]. Additionally, regarding cooked ham model mixtures, there was also a clear improvement in the PCR efficiency (from 88.0% to 94.9%). As observed, the use of a reference endogenous gene for normalization had a positive effect on the performance parameters of this system. In complex food matrices, such as meat products, the use of several ingredients and thermal treatments can affect the target gene amplification. Moreover, the DNA degradation, the presence of PCR inhibitors or the differences in the amount and quality of the extracted DNA, but also the differences in target species content can cause amplification variations. These variations in DNA yields and efficiency of amplification can be controlled by the construction of a normalized calibration curve using an endogenous control [18,27,28].

In order to validate the developed quantitative method, different blind mixtures, containing 4.0%, 2.0%, 0.8%, 0.4%, 0.2%, and 0.02% (w/w) of MPC, were used for each subset of model mixtures. Accordingly, several parameters such as trueness, precision, and robustness were assessed [22,23], and the obtained results are presented in Table 3. In terms of trueness, expressed as bias, the majority of the values are between –20.4% and 24.3%, which are within the acceptable bias of ±25.0% of the actual value over the tested dynamic range. However, there are three values that are above the criterion of acceptance, namely, samples B (82.9%), D (65.4%), and K (25.5%). Samples B and D are the raw mixtures of hams that showed, as previously mentioned, a small quantity of water after their preparation, thus affecting PCR amplifications of the target DNA and its subsequent quantification. The ice used in the sausage preparation may have affected, in a similar way, the quantification of MPC in raw sample K. The values of coefficient of variation, expressing the relative standard deviation of results, under repeatability conditions, varied between 1.3% and 24.9%, demonstrating the precision of the method over the tested dynamic range (≤25.0%). Additionally, real-time PCR runs were performed using distinct reference and blind mixtures submitted to two different thermal processes, thus confirming the robustness of the proposed quantification system (Table 3). In summary, the results of method validation and the performance parameters of real-time PCR assays clearly demonstrate the reliability and accuracy of the quantitative method.

#### 3.1.3. Effect of Food Matrix and Thermal Treatment

To evaluate the effect of thermal processing and food matrix, two distinct and independent heat treatments were used, simulating the production of cooked hams and autoclaved sausages. The preparation of both formulations was done independently and divided into two subsets, one to be analyzed without processing and the other using the corresponding thermal treatment. In the case of cooked ham, it was cooked in an oven at a temperature of 67 °C for 5 h, in controlled conditions of humidity, while the sausages were autoclaved at 121 °C for 15 min with controlled pressure (1 bar). The normalized calibration curves using MPC in ham and sausages are presented in Figure 3a and b, respectively, comparing, in both cases, the raw with thermally-processed mixtures. In Figure 3c, raw mixtures of ham and sausages are compared to evaluate the effect of food matrix. The normalized calibration curves of each set of ham mixtures practically overlapped (Figure 3a) since the slopes and b-intercepts were very close (difference of b-intercept was about 0.1 cycles), with only one significant difference at 0.1% (w/w) level. However, it can be observed that there is a decrease in the correlation coefficient of the method in raw mixtures (R^2^ = 0.951), suggesting that the excess of water present in these mixtures had a subtle negative effect on the performance of the system, as previously stated. In terms of relative LOD and PCR efficiency, the results suggest that thermal treatment had no effect on the detection of MPC since the same LOD of 0.01% (100 mg/kg) was reached in both ham systems with PCR efficiencies of 102.4% and 94.9% for raw and cooked mixtures, respectively (Figure 3a, Table 2). Regarding sausage mixtures, raw and processed (Figure 3b, Table 2), the values for PCR efficiency, slope, and R^2^ were 109.1% or 100.0%, −3.122 or −3.203, and 0.990 or 0.961, respectively. Comparing the raw with the autoclaved mixtures, a difference of about 1.4 cycles was noticed between calibration curve intercepts, which is in agreement with the significant differences found at all levels of concentration (*p* < 0.05), except for the highest one (10.0%, w/w). In this case, heat processing had a negative effect on the MPC detection since the relative LOD were 0.005% (w/w) and 0.01% (w/w), in raw mixtures and in autoclaved ones, respectively. In addition, the correlation coefficient in autoclaved sausages is slightly out of the acceptable range [22,23]. Comparing both thermal treatments in hams and sausages, the more aggressive autoclaving process seems to slightly affect the detection of MPC, but still maintaining an optimal PCR efficiency.

Food matrix clearly affects MPC detection (Figure 3c) since a difference of about 4.7 cycles between the intercepts of the calibration curves of ham and sausages is obtained, which agrees with significant differences found at all concentration levels (*p* < 0.05) and demonstrates a clear delay in the amplification of DNA from ham mixtures in relation to sausages. Moreover, the LOD of raw sausages is 0.005% (50 mg/kg, w/w) with optimal PCR performance parameters (R^2^ = 0.990, slope = −3.122, and PCR efficiency = 109.1%), contrarily to raw ham that achieved a LOD of 0.01% (100 mg/kg, w/w) and a correlation coefficient of 0.951, below the acceptable value of 0.98 (Figure 3c, Table 2). The effect of food matrix in food allergen detection by DNA-based methods has been reported by some authors [18,29,30]. The use of reference materials with different calibration models in order to simulate the real food matrices is extremely important. In addition to allowing the correct estimation and quantification of the target, it accounts with all the components of the food matrix, such as fats, carbohydrates, and other metabolites, which can interfere in DNA extraction and lead to a decrement in PCR efficiency, thus, affecting quantitative results [6,18,31]. 

### 3.2. Analysis of Commercial Samples

The applicability of the developed methods to real processed meat products (both normalized systems with processed mixtures) was done according to each type of sample, cooked ham or sausage. Thirteen commercial samples of cooked hams and sausages from distinct brands were evaluated for the presence of milk ingredients, being posteriorly analyzed by real-time PCR for quantification purposes. For comparison purposes, the same commercial hams and sausages were tested with ELISA. Table 4 presents the summarized qualitative and quantitative PCR results, as well as ELISA results, together with the relevant label information. The qualitative PCR results show that only four samples are positive to milk, namely #6, #9, #12, and #13, whose labels stated the presence of milk proteins or “may contain traces of milk”. However, in the case of sample #9, there is no label information about the presence of milk, while sample #13 clearly stated that is a “milk-free” product. All the qualitative results were confirmed by real-time PCR, with only one sample (#5) presenting negative amplification. Following our pre-established specificity criteria [19], all amplifications above 38 cycles were considered below the LOD because of the cross-reactive species. Thus, only samples #6 and #12 could be quantified for the presence of milk, obtaining 0.205% (2050 mg/kg, w/w) and 0.014% (140 mg/kg, w/w) of milk content, respectively. All the remaining samples were considered below the stipulated LOD. According to Regulation (EU) no. 1169/2011 [32], the mandatory labeling regarding potentially allergenic ingredients should be complied with. From samples whose labels stated the presence of milk protein or at traces level, 70% (5/7) were negative to milk by real-time PCR, suggesting the common precautionary labeling to comply with legislation. In the case of samples #6 and #12, which were positive to milk, the estimated amount seems to be in accordance with the label. Thus, all the tested samples were in good agreement with allergen labeling legislation [32].

ELISA and real-time PCR results seem to be well corroborated, comparing both quantitative data for four samples (#4, #6, #7, and #12) (Table 4). Samples #4 and #7 presented trace amounts of milk proteins based on ELISA (3.95 mg/kg and 8.02 mg/kg, respectively), while the results of real-PCR were <LOD, which was defined as 100 mg/kg. Sample #12 exhibited an amount of 140 mg/kg by real-PCR, which agrees with the value determined by ELISA (94.5 mg/kg). The only difference among results concerned sample #6, whose ELISA result was 10 times higher than the real-time PCR. However, considering that this sample needed a 1000-fold dilution in order to fall within the calibration curve range (2.5 to 67.5 mg/kg), the value might be overestimated. In general, these kits are specialized in evaluating trace amounts of allergenic proteins, not the quantities expressed in this specific sample (#6), which might explain the overestimated value.

## 4. Conclusions

In the present study, we propose the development of four normalized real-time PCR systems targeting the 12S rRNA mitochondrial gene of *Bos domesticus* to detect and quantify trace amounts of milk ingredients (protein concentrate) in complex meat products. Two distinct and independent thermal treatments were applied to two different meat mixtures (hams and sausages) in order to evaluate the effects of food matrix and processing on milk detection and quantification. On the one hand, the ham matrix seems to have a clear effect on milk detection since the sensitivity in hams without thermal treatment was two-fold lower than in raw sausages. On the other hand, the autoclaving process slightly decreases the sensitivity from 0.005% (w/w) in raw sausage mixtures to 0.01% (w/w) in autoclaved ones, while the soft oven-cooking treatment seems to have no effect. The developed methods were validated with blind mixtures and successfully applied to commercial meat samples. The quantitative real-time PCR results of commercial samples were further validated by ELISA, confirming the previous data. The results of commercial samples enabled the quantification of trace amounts of milk in two out of 13 samples, suggesting the excessive practice of the precautionary labeling in most of the samples.

With this study, novel, highly specific, and sensitive normalized real-time PCR systems were proposed as accurate and reliable tools for the detection and quantification of milk ingredients in meat products, which could effectively contribute to better manage milk allergens by the food industry and by the control laboratories, aiming at protecting the health of sensitized and allergic consumers.

## Figures and Tables

**Table 1:** Key data of primers and probes to target the 12S rRNA bovine gene and two universal eukaryotic regions of the nuclear 18S rRNA gene.

Primers	Sequence (5′→3′)	Amplicon (bp)	Target	Reference
916 916-R 916-P	GTACTACTAGCAACAGCTTA AGACTGTATTAGCAAGAATTGGTG FAM-TCTAGAAGGATATAAAGCACCGCCAAGT-BHQ1	121	12S rRNA	[19]
EUK-F EUK-R S5	AGCCTGCGGCTTAATTTGAC CAACTAAGAACGGCCATGCA FAM-AGGATTGACAGATTGAG-BHQ2	120	18S rRNA	[20]
18SRG-F 18SRG-R	CTGCCCTATCAACTTTCGATGGTA TTGGATGTGGTAGCCGTTTCTCA	113	18S rRNA	[21]

**Table 2:** Calibration curve parameters obtained in normalized quantitative real-time PCR systems using model mixtures of MPC in ham and sausages, with and without thermal treatment.

Parameter	Ham		Sausages	
	Raw	Cooked	Raw	Autoclaved
Correlation coefficient (R^2^)	0.951	0.980	0.990	0.961
Slope	−3.265	−3.450	−3.122	−3.203
PCR efficiency (%)	102.4	94.9	109.1	100.0
Relative LOD (%)	0.010	0.010	0.005	0.010

**Table 3:** Validation results of normalized quantitative real-time PCR systems applied to blind mixtures of MPC in ham and sausages.

Samples	Milk (% w/w)		SD ^2^	CV ^3^ (%)	Bias (%) ^4^
	Actual	Mean Predicted ^1^			
Raw ham					
A	4	4.70	0.41	8.7	18.2
B	0.8	1.46	0.12	8.1	82.9
C	0.4	0.46	0.08	16.1	15.8
D	0.2	0.07	0.02	16.0	−65.4
Cooked ham					
E	4	4.50	0.59	1.3	−11.6
F	0.8	0.70	0.12	17.4	−10.8
G	0.4	0.40	0.09	23.7	−5.0
H	0.2	0.17	0.04	19.7	−14.5
Raw sausages					
I	2	1.59	0.30	19.1	−20.4
J	0.4	0.48	0.05	9.5	21.0
K	0.2	0.15	0.03	21.8	−25.5
L	0.02	0.023	0.006	24.9	−15.20
Cooked sausages					
M	2	1.63	0.23	14.4	−18.5
N	0.4	0.46	0.09	19.9	14.8
O	0.2	0.17	0.04	24.5	−15.0
P	0.02	0.025	0.005	19.8	24.3

^1^ Mean values of replicate assays (*n* = 8) of two independent runs; ^2^ SD, standard deviation; ^3^ CV, coefficient of variation; ^4^ Bias = (mean value − true value)/true value × 100.

**Table 4:** Results of the application of qualitative PCR, normalized quantitative real-time PCR and ELISA to detect and quantify milk ingredients in commercial samples of cooked hams and sausages.

Samples	Relevant Label Information	Qualitative PCR		Real-Time PCR			ELISA
		18SRG-F/18SRG-R	916-F/916-R	EUK-F/EUK-R (Ct ± SD) ^1^	916-F/916-R (Ct ± SD) ^1^	Estimated Milk Content (mg/kg) (mean ± SD)	Estimated Amount (mg/kg) (mean ± SD) ^2^
Cooked hams (from pork)							
1	Milk proteins	+ ^3^	- ^4^	26.60 ± 0.16	40.76 (1/3) ^5^	<LOD ^6^	<LOQ ^7^
2	No information about milk	+	-	29.57 ± 0.28	39.77 (1/3)	<LOD	<LOQ
3	No information about milk	+	-	26.83 ± 1.21	45.58 (1/3)	<LOD	<LOQ
Cooked hams (from turkey)							
4	May contain traces of milk	+	-	21.88 ± 0.62	41.15 ± 1.10 (2/3)	<LOD	3.95 ± 0.10
5	May contain traces of milk	+	-	25.38 ± 0.08	(0/3)	ND ^8^	<LOQ
6	Milk proteins	+	+	19.56 ± 0.04	34.19 ± 0.27 (8/8)	2050 ± 320	23300 ± 4722^ 9^
Sausages (from pork)							
7	No information about milk	+	-	23.75 ± 1.39	40.52 ± 0.90 (3/3)	<LOD	8.02 ± 2.06
8	No information about milk	+	-	21.31 ± 1.80	40.35 ± 0.27 (2/3)	<LOD	<LOQ
9	No information about milk	+	+	24.14 ± 0.40	39.99 ± 0.85 (3/3)	<LOD	<LOQ
Sausages (from turkey)							
10	May contain traces of milk	+	-	22.49 ± 0.17	39.90 (1/3)	<LOD	<LOQ
11	May contain traces of milk	+	-	28.67 ± 0.14	41.91 ± 2.07 (3/3)	<LOD	<LOQ
12	May contain traces of milk	+	+	18.67 ± 0.01	35.77 ± 0.60 (8/8)	140 ± 30	94.5 ± 7.78^ 9^
13	Without milk addition	+	+	20.05 ± 0.04	41.01 ± 1.90 (2/3)	<LOD	<LOQ

^1^ Mean values of cycle threshold (Ct) ± standard deviation (SD) (*n* = 8) of two independent runs; ^2^ mean values (mg/kg)±standard deviation (SD) of replicate assays (*n*=3); ^3^ Positive amplification (+); ^4^ No detectable amplification (-); ^5^ positive replicates and total number of replicates ^6^ <LOD (100 mg/kg); ^7^ <LOQ (2.5 mg/kg); ^8^ ND, not detected; ^9^ estimated amounts obtained from previously diluted protein extracts.
